# Exome-Sequencing Confirms *DNAJC5* Mutations as Cause of Adult Neuronal Ceroid-Lipofuscinosis

**DOI:** 10.1371/journal.pone.0026741

**Published:** 2011-11-04

**Authors:** Bruno A. Benitez, David Alvarado, Yefei Cai, Kevin Mayo, Sumitra Chakraverty, Joanne Norton, John C. Morris, Mark S. Sands, Alison Goate, Carlos Cruchaga

**Affiliations:** 1 Department of Psychiatry, Washington University, St. Louis, Missouri, United States of America; 2 Department of Pediatrics, Washington University, St. Louis, Missouri, United States of America; 3 Department of Neurology, Washington University, St. Louis, Missouri, United States of America; 4 Hope Center Program on Protein Aggregation and Neurodegeneration, Washington University, St. Louis, Missouri, United States of America; 5 Department of Genetics, Washington University, St. Louis, Missouri, United States of America; University of Edinburgh, United Kingdom

## Abstract

We performed whole-exome sequencing in two autopsy-confirmed cases and an elderly unaffected control from a multigenerational family with autosomal dominant neuronal ceroid lipofuscinosis (ANCL). A novel single-nucleotide variation (c.344T>G) in the *DNAJC5* gene was identified. Mutational screening in an independent family with autosomal dominant ANCL found an in-frame single codon deletion (c.346_348 delCTC) resulting in a deletion of p.Leu116del. These variants fulfill all genetic criteria for disease-causing mutations: they are found in unrelated families with the same disease, exhibit complete segregation between the mutation and the disease, and are absent in healthy controls. In addition, the associated amino acid substitutions are located in evolutionarily highly conserved residues and are predicted to functionally affect the encoded protein (CSPα). The mutations are located in a cysteine-string domain, which is required for membrane targeting/binding, palmitoylation, and oligomerization of CSPα. We performed a comprehensive *in silico* analysis of the functional and structural impact of both mutations on CSPα. We found that these mutations dramatically decrease the affinity of CSPα for the membrane. We did not identify any significant effect on palmitoylation status of CSPα. However, a reduction of CSPα membrane affinity may change its palmitoylation and affect proper intracellular sorting. We confirm that CSPα has a strong intrinsic aggregation propensity; however, it is not modified by the mutations. A complementary disease-network analysis suggests a potential interaction with other NCLs genes/pathways. This is the first replication study of the identification of *DNAJC5* as the disease-causing gene for autosomal dominant ANCL. The identification of the novel gene in ANCL will allow us to gain a better understanding of the pathological mechanism of ANCLs and constitutes a great advance toward the development of new molecular diagnostic tests and may lead to the development of potential therapies.

## Introduction

The neuronal ceroid lipofuscinosis (NCLs) are the most common group of inherited neurodegenerative diseases in children, with an incidence in the U.S. of approximately 1 in 12,500 live births [1]. NCLs encompass a genetically heterogeneous group of disorders, clinically characterized by progressive deterioration of cognitive and motor skills, visual impairment, and premature death [2]. The onset of the clinical symptoms in addition to the differences in ultrastructural features of the lipopigment inclusions underlie the nosological spectrum of the NCLs: infantile (INCL, Santavuori-Haltia, MIM 256730), late-infantile (LINCL, Jansky-Bielschowsky, MIM 204500), juvenile (JNCL, Batten disease, Spielmeyer-Vogt, MIM 204200), adult (ANCL, Kuf's disease, MIM 204300), and Northern epilepsy (NE, progressive epilepsy with intellectual disability) [2].

Over the last fifteen years, at least 300 mutations in ten genes have been associated with NCLs such as *CLN1* (PPT1 (MIM 256730)), *CLN2* (TPP1 (MIM 204500)), *CLN3* (MIM 204200), *CLN5* (MIM 256731), *CLN6* (MIM 601780), *CLN7* (MFSD8 (MIM 610951)), *CLN8* (MIM 600143), *CLN10* (CTSD (MIM 610127)), *CLCN6* (MIM 602726) and *SGSH* (MIM 605270) (NCL Mutation Database, URL). This genetic progress has led to the development of molecular testing tools and promising rational therapeutic approaches [3]. However, there is no cure for NCLs, and treatments are limited to palliative care.

Adult onset NCLs (ANCLs) represent between 1.3% and 10% of NCLs cases [4]. ANCLs are rapidly worsening conditions with a wide range of age at onset (6–62 yr) and broad clinical variability. Two main clinical subtypes have been described: progressive myoclonus epilepsy (type A), and dementia with motor disturbances, such as cerebellar, extrapyramidal signs, and dyskinesia (type B). However, there is some overlap, or a continuum of signs between the two types, particularly late in the course of the disease. Therefore, it is not always easy to differentiate them [4]. Unlike other NCLs there is an absence of retinal degeneration [5]. Pathologically, the ceroid-lipofuscin accumulates mainly in neurons and contains subunit C of the mitochondrial adenosine-triphosphate synthase (SCMAS) [6], but has different ultrastructural appearances such as granular osmiophilic deposits (GRODs) and fingerprint, curvilinear, or rectilinear structures [7].

ANCLs are genetically heterogeneous with either a sporadic, autosomal recessive (Kufs' disease, MIM 204300) or dominant (Parry's disease, MIM 162350) pattern of inheritance in confirmed cases [4], [5]. Three known NCL genes were previously associated with atypical ANCLs such as *PPT1* [8], [9], *CLN5*, and *N-Sulfoglucosamine Sulfohydrolase* (*SGSH*, MIM 605270) [10], suggesting that some of the ANCLs were not distinct genetic entities and raising the possibility that they actually represent an extreme of a clinical spectrum of low penetrance and variable expressivity of NCL mutants [10], [11]. This knowledge, although significant, could not contribute to molecular elucidation of the ANCLs.

**Figure 1:**
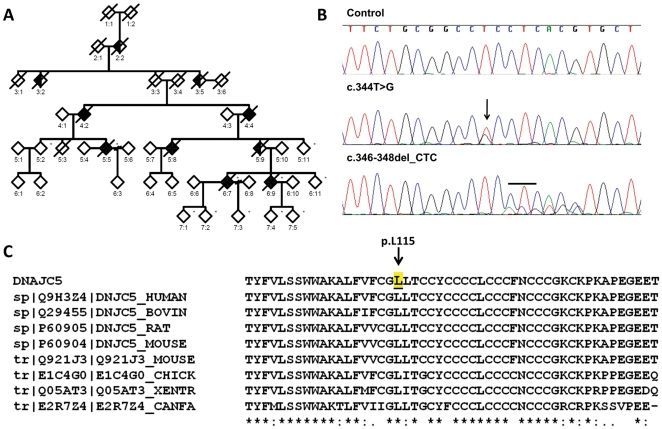
Pedigree of ANCL family, Sanger sequencing results and Multiple Alignment of CSPα. (A) Pedigree of the ANCL family. Black symbols denote affected individuals; open symbols denote unaffected individuals. (B) Chromatograms of exon 4 of *DNAJC5* gene, showing sequences of identified heterozygous mutations. (Upper panel) Sequence of an unaffected elderly control, (middle panel) sequence showing heterozygous mutation c.344T>G in the affected proband, (lower panel) sequence showing heterozygous mutation c.346_348delCTC in affected from a second family (Validation set). Arrow and black short line indicate the position of the missense and deletion mutation, respectively. (C) Multiple Sequence alignment of Cysteine-string domain of CSPα amino acid sequence among homologous genes in mammals. pL115 is highlighted in yellow and indicated by the arrow.

That was the state of the art when we started to perform an exome-sequencing study in the largest family (10 affected members over 5 generations) with autosomal-dominant ANCLs, known until now (Figure 1A) [12]. However, during the course of this study, two genes, a well known NCL gene (*CLN6*) [11] and *DNAJC5* gene (MIM, 611203) [13] have been associated with autosomal recessive (locus NCL4A) and dominant (locus NCL4B) cases of ANCLs, respectively. There was no apparent correlation of the underlying genetic defect with the clinical course and the ultrastructural features between the studies and, to date, neither of them has been independently replicated. Independent replication studies are important especially in ANCLs, wherein a critical evaluation of the literature led to the rejection of 68 (out of 118) cases published as Kufs' disease [5], which means that there is a high rate of misdiagnosis in ANCLs. These results also indicate that the genetic architecture of ANCL is more varied and complex than previously thought.

In this study, we performed exome-sequecing in three family members from a family with autosomal dominant ANCL with early dementia [12] (Figure 1A). Several recent studies have successfully identified the disease-causing genetic variant in non-NCL diseases with similar pedigrees by exome-sequencing [14]. Exome-sequencing is a very powerful technique, especially in families that are not big enough for classical linkage studies. In previous studies, the pathogenic variant was identified by only sequencing as low as two or three individuals [15].This method is a hypothesis-free approach that allows for a targeted enrichment and resequencing of nearly all exons of protein-coding genes. Protein-coding exons account for only 1% of the human genome, but 85% of Mendelian diseases are caused by mutations in this genomic space.

## Results

### Exome Sequencing

Overall, a mean of 158.6 million reads were generated for the three samples. Approximately 80% of these were aligned to the human reference genome (hg18) and 95% of these fell onto targeted and enriched exons. Reads not corresponding to the targeted bases of the exome were discarded (less than 2%). The mean exome coverage was 94.7% with >5× fold coverage. After filtering for a minimum length good quality sequence of 30 and a minimal sequence read depth of 5× (standard parameters), we identified on average 38,179±84 coding single nucleotide substitutions (SNSs) at a transitions-to-transversions ratio of 3.05, and 2931±10 small insertions and deletions (indels), among all three individuals.

To identify pathogenic variants, we consecutively filtered these variants by subjecting them to an analytical pipeline for high-confidence variant calling and annotation. Briefly, we discarded (a) common and known variants present in HapMap, the dbSNP130 Database or 1,000 Genomes Project at a frequency of greater than 5% for heterozygous and 30% for homozygous calls, and (b) variants in intergenic or intronic regions [16]. After filtering, 2,337±102 SNPs and 210±2 indels were identified as novels. Next, we further focused on functionally significant variants such as missense, nonsense, or splice-site changes, with sufficient and consistent depth and quality values plus a high rate of concordance among the three samples. Thus, we identified 96 SNSs (95 missense, 1 nonsense), and 13 indels which were private variants for these members of this family (Table 1). There were 24 heterozygous non-synonymous coding variants and three heterozygous indels (Table 1) that were present in both affected individuals but absent in the control. In order to remove systematic artifacts and rare variants, we checked these variants against an additional Washington University exome database of 59 individuals, which reduced the list to 19 variants and three indels (Table 2). These 22 high-quality variants had a median SNP quality score of 228 (range 61 6095) and a read depth of 124.5 (range 9–381), and were selected for validation using two different genotyping technologies: the MassARRAY SNP (Sequenom, Inc) and KASPar v4.0 SNP (KBioscience) genotyping systems [17], [18], [19]. Only one variant turned out to be a false positive, which was most likely due to a mapping error. Thus, we have a low rate of false discovery of 0.045.

**Table 1:** Filtering process of coding single nucleotide substitutions.

Sample	Total Coding SNSs	Non-synonymous SNSs¶	SNSs after filtering	SNSs in both cases	SNSs unique to cases	SNSs after removing found in Wash U exomes
Affected	38142	9202	674	96	24	19
Affected	38276	9285	636			
Control	38120	9177	711			
Percent of SNSs remaining§	100	24.1	1.8	0.25	0.06	0.04

¶dbSNP, HapMap and 1000 Genomes commons variants were removed (het<5%, hom<30%).

§ Percentage of SNSs after each step.

**Table 2:** Summary of exome sequencing results from ANCL.

Chr	Position (bp)	Ref Base	Variant	Gene	AA Subs.	Type of Change	Polyphen2	SeggPat
1	16,915,434	T	C	NBPF1	K135E	missense	Benign	No
2	130,899,804	T	C	CCDC74B	H149R	missense	Benign	No
3	64,072,900	*	−AA	LOC100287879	None	frameshift	Not predicted	No
3	33,883,492	G	A	PDCD6IP	G429S	missense	Probably damaging	Yes
3	75,788,010	A	C	ZNF717	V255G	missense	Benign	No
4	155,191,162	G	A	DCHS2	T1701I	missense	Possibly damaging	No
4	164,247,234	C	G	NPY1R	G158A	missense	Possibly damaging	No
4	148,653,570	A	G	ARHGAP10	I40V	missense	Benign	No
4	162,421,268	A	G	FSTL5	M452T	missense	Benign	No
5	180,376,974	T	G	BTNL8	H311Q	missense	Probably damaging	No
6	132,030,966	G	C	ENPP3	L398V	missense	Unknown	No
7	100,550,039	*	+CTC	MUC3A	None	coding	Not predicted	No
10	90,356,030	*	−TTTTA	LIPJ	None	splice-3	Not predicted	Yes
13	19,042,781	T	C	LOC729501	T434C	missense	Unknown	No
13	115,012,439	A	G	CDC16	M311V	missense	Benign	No
13	29,287,552	C	T	SLC46A3	V109I	missense	Benign	No
15	43,038,399	C	T	TTBK2	R1110H	missense	Possibly damaging	No
16	16,218,686	G	A	ABCC1	V1211I	missense	Possibly damaging	No
16	25,123,254	C	G	LCMT1	S17T	missense	Probably damaging	No
16	24,804,954	A	T	TNRC6A	Q1112H	missense	Probably damaging	No
20	62,562,226	T	G	DNAJC5	L115R	missense	Possibly damaging	Yes
X	3,747,372	C	T	LOC389906	W263*	nonsense	Unknown	No

Chr: chromosome; Position (bp) is according to Human Genome build 37 (hg37); Reference base: reference allele according to hg37; Variant: nucleotide found by exome sequencing; Gene: official Symbol provide by HGNC; AA Substitution: amino acid change resulting from the observed variant; Type of change: predicted change in the gene sequence; Protein prediction: based on Polyphen analyses of the predicted effect of the substitution on protein function; Segregation pattern: present in all affected tested.

### Identification of the causative variant

Next, we carried out an extended co-segregation analysis within the family for all the validated SNPs. In total, we genotyped three affected samples and three elderly healthy individuals (See introduction methods). As a result, only two SNSs, one located in *PDCD6IP* (Programmed cell death 6-interacting protein, MIM 608074) and one in *DNAJC5*, plus a deletion in the *LIPJ* gene (lipase-like, ab-hydrolase domain containing 1, MIM 613921) segregated perfectly with disease status (Table 2).

It has been shown that disease genes display significant functional clustering in molecular networks [20], [21]. One-third of known disorders with two or more associated genes were found in physical clusters of genes with the same phenotype [20]. Therefore, we used a disease-network approach using all NCL genes as a training group (see introduction methods) to prioritize further validation among these three genes (*PDCD6IP, DNAJC5 and LIPJ*). Surprisingly, we found that these three variants were in the top five genes in the combined analysis (See: Table S1), suggesting that they may be functionally or structurally related with NCLs encoded genes and constituting true candidates as ANCLs causative genes. We also noticed that out of these three variants, *PDCD6IP* and *DNAJC5* have the highest Genomic Evolutionary Rate Profiling (GERP) and are predicted to be damaging for the protein (Table 2). The deletion in the *LIPJ* gene was predicted to remove a potential donor splice site by using the Human Splicing Finder server (Consensus Values (CV) for wild type 69.84 and 36 for the mutant, an average reduction (-ΔCV) of 48.45) [22]. Taking into account this evidence, *PDCD6IP, DNAJC5 and LIPJ* were all identified as excellent plausible candidates for the genetic defect responsible for ANCL. Therefore, we performed genotyping for each variant in a cohort of 1,600 (3,200 chromosomes) ethnically matched controls. The p.G429S variant in the *PDCD6IP* gene and the deletion on the 3′-splicesite in the *LIPJ* gene turned out to be rare variants with a MAF of 0.01 and 0.03, respectively.

The only variant remaining is a single nucleotide substitution (c.344T>G) that causes a p.L115R amino acid change in *DNAJC5* gene. Sanger sequencing was performed to confirm the presence of the mutation in *DNAJC5* gene in all affected individuals in the family (Figure 1B). We also analyzed the exome-sequencing data of all individuals for mutations in previously reported genes associated with ANCLs such as *PPT1*, *CLN5*, *SGSH* and *CLN6* [8], [9], [10], [11] and revealed no non-synonymous changes in any of them.

The fact that the c.344T>G variant in *DNAJC5* was present in all the affected individuals but not in 1,600 control individuals strongly indicates that this is the underlying genetic cause of the ANCL phenotype in this family.

In order to confirm that mutations in the *DNAJC5* gene causes ANCL, we used Sanger sequencing to analyze the entire coding sequence plus the exonic flanking region in three other independent autosomal dominant familial cases of ANCL and one of LINCL (internal validation set). We found an in-frame single codon deletion (c.346_348 delCTC), which causes a deletion of p.L116del (Figure 1B) in a second family. We designed a Kaspar assay to test this deletion, and did not detect it in more than 3,200 chromosomes from our control samples. None of these variants have been previously reported in dbSNP (build 134) or 1000 genomes project database (20101123 releases).

Thus, combining all the genetic analysis together; we found that different novel mutations in *DNAJC5* are present in unrelated families and exhibit perfect segregation with disease status. These variants are located in highly conserved regions (GERP score of 5.34), are predicted to be damaging and are not present in 1,600 controls. Therefore, our results replicate the recent report of mutations in *DNAJC5* gene as a cause of ANCL [13].

### Bioinformatic analysis

*DNAJC5* is located at 20q13.33 with genomic coordinates: 62,526,454–62,567,383 (GRCh37) and encodes a 198-amino acid residue protein (cMW 22.1 kDa) called CSPα for cysteine-string protein-a (NP_079495.1). CSPα contains three highly conserved domains: the N-terminal J-domain (15–83 aa), the linker region (84–112), and the cysteine string domain (CSD) (113–136 aa) [23].

The p.L115R and p.L116del mutations affect highly conserved dileucine residues located immediately N-terminal to the CSD (Figure 1C); this region is responsible for palmitoylation, membrane binding/targeting and oligomerization of CSPα [24], [25], [26]. Therefore, we took advantage of the existence of robust *in silico* tools (See introduction methods), which are widely validated with curated experimental data, to test the functional impact of the mutations on these processes.

### Palmitoylation Analysis

**Figure 2:**
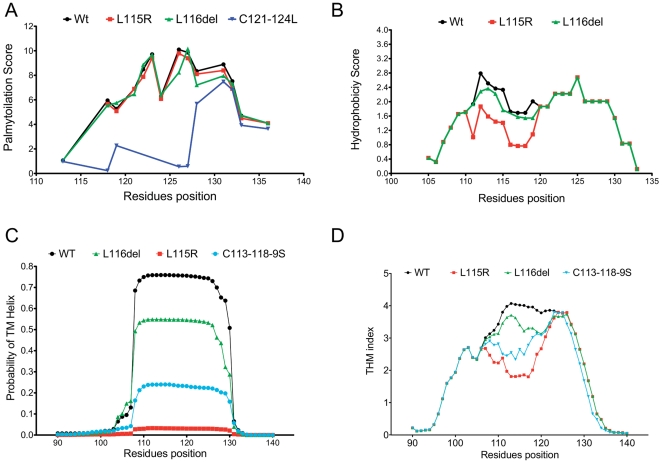
Palmitoylation, Hydrophobicity and membrane binding profile of CSPα. (A). *In silico* predicted palmitoylation profile of cysteine-string domain in wild-type, p.L115R, p.L116del and positive control (C121-124S). (B) Intrinsic hydropathy plots of CSD amino acid sequences in wild-type, p.L115R and p.L116del(C) The posterior probability for transmembrane helix for the wild-type, p.L115R, p.L116del and positive control (C113-118-9S). (D) Transmembrane profile amino acid sequences of wild-type, p.L115R, p.L116del and positive control (C113-118-9S). Wild-type (Black Line), p.L115R (Red Line), p.L116del (Green Line) and positive controls (Blue line).

First, we performed an *in silico* analysis of the impact of the mutations on the palmitoylation status of CSPα. As shown in Figure 2A, changes in the p.C121-124L residues used as a positive control, showed a significant reduction in the levels (3.217±2.723) of predicted palmitoylation compared to the wild type (WT) (6.942±2.579) (p<0.05 kruskall-wallis test), which is in agreement with experimental data [25], [26]. In contrast, we did not find any significant (p>0.05) change in the pattern of palmitoylation induced by p.L115R (7.093±1.919) and p.L116del (6.677±2.395) over the wild type sequence, although, the p.L115R mutation eliminates palmitoylation of p.C113.

### Hydrophobicity Profile of CSPα

The CSD is a highly hydrophobic region, and residues in the N-terminal half, are likely to play a key role in membrane association [25], [26]. The general index of hydrophobicity (mean ± sd, for the segment from 110–120 residues) for WT is 1.699±0.69, for p.L115R is 1.181±0.65, and for p.L116del is 1.559±0.65 (Figure 2B). A nonparametric ANOVA (kruskal-wallis statistic), followed by Dunn's multiple comparison test showed that there is a significant reduction in the global hydrophobicity index induced by the p.L115R mutation (p<0.05) but is not significant for the p.L116del mutation.

### Membrane binding/targeting analysis

Next, we evaluated *in silico* the propensity of CSPα to interact with the lipid bilayer and the impact of the mutations on the hydropathy of the CSD. We measured the free energy of transfer from water to a phosphocholine bilayer interface of CSD. In this thermodynamic free energy scale, the stable TM helical segments show favorable water-to-membrane transfer free energies (ΔG<0). We found that WT CSPα has a ΔG −5.69 kJ/mol. while the mutations p.L116del (ΔG −5.13 kJ/mo) and p.L115R (ΔG −4.32 kJ/mo), reduce this propensity by similar amounts to the positive control p.C113-118-9S (ΔG −4.58 kJ/mo). This result suggests that the mutated domain will release less energy to bind to the membrane than the WT. In addition, we examined the difference between the octonal and interface scales (Δ*G_woc_*−Δ*G_wif_*); a measure which identifies segments that tend to prefer a transbilayer helix conformation relative to an unfolded interfacial location. The wild type sequence showed ΔG 6.55 kJ/mol, while p.C113-119S (ΔG 6.88 kJ/mol), p.L116del (ΔG 7.24 kJ/mol) and p.L115R (ΔG 8.24 kJ/mol) reduced this propensity. This finding suggests the mutated domain alters CSPα such that it would prefer to be in water than affiliated to the membrane. The results of our simulation are in agreement with the weak affinity for the membrane reported for the CSD of CSPα [25], [26]. In addition, these results suggest that the mutations reduce the intrinsic tendency of CSPα to bind to the membrane.

In addition, both p.L115R (0.03036±0.001724) and p.L116del (0.5190±0.05983) reduce the probability of CSPα forming membrane-binding domains compared to WT (0.7369±0.03764), and exhibit values similar to the positive control p.C113-116S (0.2269±0.01267) (Figure 2C–D). Thus, the number of residues that were expected to be part of the membrane-binding domain decreased from 18 residues for WT to less than one in p.L115R, 12 residues for p.L116del, and 5 residues for the positive control (p.C113-116S). This result suggests that the mutations are dramatically reducing what has been experimentally demonstrated to be the minimum-required membrane-binding domain [25], [26].

### Oligomerization Analysis

We employed the prediction of amyloid structure aggregation (PASTA) software to identify the regions of CSPα, which are likely to stabilize the CSPα-CSPα dimers into aggregates [27]. We found that the region between p.F110-P138 residues has a strong propensity to assemble into antiparalell ß-sheets (See: Figure S1). The same core region for oligomerization identified experimentally [24], [28]. On average this segment has a pairing energy score of −25 in WT, −25.4 in p.L115R, and −25.3 in p.L116del mutations supporting the antiparalell assembly. In addition, we found that on average the aggregation propensity [h(k)] of WT CSD is around 0.04 as well as p.L115R (0.04) and p.L116del (0.04). The positive control (Aβ-40) has an aggregation propensity around 0.05 (See: Figure S1) [27].

**Figure 3:**
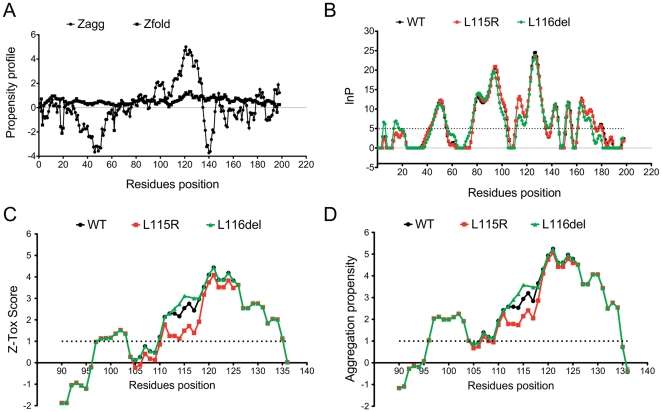
Aggregation profile of CSPα. (A). Intrinsic aggregation (Zagg Score) and folding propensity (Zfold score) profile of amino acid sequence of CSPα Wild type. (B) Predicted protection factors (lnp score) of wild-type, p.L115R and p.L116del amino acid sequence. (lnp<5 for nonprotected residues; lnp>5 for protected residues, dashed straight line) (C) Toxicity profile (Ztox score) of cysteine-string domain of wild-type, p.L115R and p.L116del amino acid sequence. High toxic propensity (Zagg>1, dashed straight line). (D) Aggregation propensity (Zagg score) profile of cysteine-string domain of wild-type, p.L115R and p.L116del amino acid sequence region. High intrinsic aggregation propensity (Zagg>1, dashed straight line). Wild-type (Black Line), p.L115R (Red Line) and p.L116del (Green Line).

Due to the lack of sufficient structural data of this region of CSPα (only the tertiary structural information for residues (1–109) at the N-terminal region of the mouse homologue of CSPα is currently known), it was not possible to accurately simulate the effect of the mutations at a structural level, and thus, we performed an initial analysis assuming an unfolded state. In general, CSPα has a tendency to be unfolded, as is shown in Fig. 3A by the Z-fold score [29]. Next, we calculated the intrinsic aggregation propensity of CSPα, initially assuming a model from unfolded to fibrillar state [30]. We found that between p.F110-N130 residues, there is a region with a high propensity to aggregate (Zagg score >1): WT (Zagg score: 3.320±1.182) (Figure 3A) [31]. This intrinsic propensity is slightly higher but not significant for the mutation p.L116del (Zagg score: 3.401±1.180) and slightly lower for p.L115R (Zagg score: 3.041±1.282) (Figure 3D). In addition, the propensity to form protofibrillar species (Ztox score) is also found to be high in this region of WT (Ztox score: 2.787±0.8957), p.L115R (Ztox score: 2.384±0.9925) and p.L116del (Ztox score: 2.867±0.8973) (Figure 3C). There is a high correlation (r = 0.97) between Ztox score and Zagg score for the region between p.F110-N130, which supports the idea that the CSD of CSPα forms stable fibrillar species.

However, in a folded state, regions that are highly aggregation prone are protected from aggregation because they are buried within the native structure without exposure to the solvent and hence, unavailable for intermolecular interactions [32]. It is also crucial to note that it has been suggested CSD may adopt a helical or beta sheet structure that aligns CSPα parallel to the plane of the membrane [26], possibly allowing CSPα to adopt a folded state. Therefore, we tested the propensity of CSPα to be protected; and found that this region p.F110-N130 may be protected in WT (14.07±6.110), p.L115R (13.97±5.743) and p.L116del (12.07±6.421) (lnp<5 for nonprotected residues; lnp>5 for protected residues) [32] in a folded state (Figure 3B).

In summary, an *in silico* analysis indicates that the p.L115R and p.116del mutations are not directly affecting the palmitoylation or the propensity of CSPα to form aggregates per se, but by modifying the membrane-targeting sequence, the physical and chemical properties of CSPα are altered, weakening the membrane binding and consequently affecting intracellular sorting of CSPα.

There are potential errors and biases in the assumptions *in silico* analysis takes into account, therefore these results should be interpreted cautiously. We used as proof of principle, controls with the strongest experimental support available to validate our findings. Nevertheless, further experimental studies are needed to understand the pathogenicity of these mutations.

## Discussion

We have confirmed that mutations in *DNAJC5* cause autosomal dominant ANCL [13]. However, ANCLs are disorders clinically and genetically very heterogeneous [4]. The subsequent difficulty in performing an accurate diagnosis had contributed as a limiting factor in the identification of its genetic cause [5]. However, to date, two different groups have been able to concurrently and independently identify the same *DNAJC5* gene and same mutations using different and complementary approaches, which consolidate and validate the results [13](shown here). However, mutations in *DNAJC5* are currently explaining approximately 25% of the autosomal dominant ANCLs [13] Therefore, It is possible that other forms of ANCLs may have another genetic cause.

This is the first replication study of the identification of mutations in the *DNAJC5* gene in ANCLs. By performing whole-exome sequencing in a multigenerational family with autosomal dominant ANCL (Figure 1A), we have identified a novel single-nucleotide variation (c.344T>G) in *DNAJC5*. In addition, using Sanger sequencing we found an in-frame single codon deletion (c.346_348 delCTC) in an independent family (Figure 1B). Thus, these variants fit genetic criteria for disease-causing mutations: they are present in unrelated families (Figure 1B); they exhibit perfect segregation with disease status (Table 2), they are not present in any healthy controls tested, they are located in evolutionarily highly conserved residues (Figure 1C), and they are predicted to functionally affect the encoded protein (CSPα) (Table 2).

Whole exome sequencing has proved useful to identify the pathogenic variant in monogenic disease [14], [15]. It is a rapid and cost-effective method that only requires sequencing in a small number of individuals. To date, different approaches to whole exome sequencing have been used including various designs, filtering methodologies and analytical strategies [15]. Some examples are sequencing several affected individuals from different families, sequencing two affected individuals from the same family, and combining whole-exome sequencing data with linkage data, among others [13], [14], [15]. Our design included two distantly-related affected individuals and a healthy sibling of one of the affected (and first cousin of the other affected individual) (Figure 1A). By selecting the two most distantly-related affected individuals available, the number of non pathogenic variants due to relatedness is dramatically reduced (Table 1).

In fact, 674±38 novel non-synonymous variants were found in each sample, but only 95 were shared by the two affected individuals. Subtracting the variants in common between the control and affected individuals, 25 SNSs were remaining (Table 1). Taking into account that on average 50% of genetic variants are shared between siblings and 12.5% between first cousins, we expected approximately 40 SNSs to remain after filtering against the control. To control for the false positive variants due to technical errors associated with Next-Generation Sequencing Platforms, all the three samples were run in the same flow cell. Thus, it is likely that the number of shared variants by both affected individuals was initially overestimated, and these artifacts were then filtered out by comparing with the control.

As done in other studies [15], additional affected family members were genotyped to identify the variants that are present in all of the affected individuals. A major challenge of whole-exome sequencing is to uniquely identify the causative variant. Most of the current approaches are based on first, removing candidate variants (non-synonymous, non-sense and splice-site variants) that are present in public databases (1000 genomes project and dbSNP), and second, on selecting only the variants present in the affected individuals. As a result, we found three unique variants located in the *PDCD6IP*, *LIPJ* and *DNAJC5* genes, which were present in all the affected family members but not in public databases. Interestingly, these three genes also exhibited the highest values based upon their GERP scores (Table 2). In order to elucidate the real cause of ANCL in this family, a large series of population controls were screened to verify if these variants in the PDCD6IP and LIPJ genes were present in healthy individuals. Only 16 and 8 heterozygous carriers for the variants on the *PDCD6IP* and *LIPJ* genes were found, respectively. It is important to note that these variants were not present in the 1000 Genomes Project or in the dbSNP Database at the time of the analysis. Similar to others, [14], [15] this study clearly shows that pathogenic or causative variants can be identified by performing exome-sequencing in a small number of family members. It is now clear that by selecting two affected family members distant in the pedigree and one unaffected sibling of one of the affected individuals, and by running the three samples simultaneously, the number of potential candidate variants is significantly decreased. Our study also shows that although the public databases are very useful in removing commonly found variants (Table 1), they are still not comprehensive enough to eliminate all possible rare variants and unequivocally identify the causative mutation. Most importantly, screening a large number of non-affected individuals is still necessary.

*DNAJC5* gene encodes CSPα, which is a key element of the synaptic molecular machinery and accounts for 1% of all vesicle proteins [33], [34], as well as part of the general exocytotic machinery [35]. The synaptic vesicle localization and chaperone activity of CSPα suggests that it may function in rescuing synaptic proteins that have been unfolded by activity-dependent stress [36]. Deletion of CSPα in flies and mice results in neurodegeneration and impairs synaptic function [36], [37], [38], [39]. CSPα has mostly been found associated with vesicles; however, it has a weak membrane affinity. Furthermore, there is an inverse correlation between membrane targeting of CSPα palmitoylation and adequate intracellular trafficking [25], [26]. Site-directed mutations that enhance membrane association such as p.C121-124L prevent adequate palmitoylation and lead to accumulation of CSPα in the ER and Golgi apparatus [25], [26]. In contrast, residue changes such as p.C113-119S (p.L115R, p.L116del, shown here in Figure 2C–D) reduce binding to the membrane resulting in inadequate palmitoylation. They exhibit a localized punctuated pattern throughout the cytoplasm [25], [26], co-localize with markers of ER–Golgi intermediate complex (Golgi SNARE proteins), and show a significant reduction in synaptic regions [13].

A potential neuronal-specific effect of this disproportionate and persistent CSPα missorting is a depletion of CSPα [13] and possibly some of its SNARE partners the synapse, leading to a disruption in neurotransmission and synaptic dysfunction as displayed by the CSPα null animal models (mimicking loss of function) [36], [37], [38], [39].

CSPα self-associates, forming oligomers [24], [28], [40]. The pG83–C136 residues constitute the core region for CSPα oligomerization [24]. Our analysis revealed that a more localized region between residues pF110-P138 has a tendency to form antiparallel ß-sheets species (See: Figure S1), consistent with reports of the CSPα-CSPα dimers that are stable to temperature- and SDS-resistant particle [28], [40]. We did not find any significant increase in the intrinsic tendency of the mutations to aggregate (Figure 3C–D). However, the unattached form of the protein can induce conformational changes that facilitate a more reactive oligomeric state (Figure 3A, D). The effective increase in concentration of soluble CSPα produced by these mutations can also increase the level of macromolecular crowding, which in turn may dramatically enhance the own propensity of CSPα to aggregate, as has been shown for α-synuclein [41].

In a macromolecular crowded environment, the equilibrium between protein folding and protein-protein interactions is driven towards the lower volume (folded to unfolded) species [42]. CSPα has been shown to have a high affinity for unfolded proteins [23]. In a crowded environment, this can increase the likelihood of CSPα to interact with itself (more stable CSPα-CSPα dimers can be generated) and with other amyloidogenic partners such as α-synuclein [38], [43]. Recently, it has been shown that CSPα-CSPα dimers appear to be the main form of CSPα found in the brains of carriers of the ANCLs mutation [13].

Another potential target of abnormal interactions of CSPα are the synaptic proteins, especially those which are central to synaptic vesicles exocytosis, including proteins from the v- and t-SNAREs complex, and the putative Ca2+ sensor synaptotagmin 1, which undergoes palmitoylation in the golgi [44]. Abnormal CSPα-CSPα dimers may impede appropriate synaptic vesicle targeting and subsequently, disrupt neurotrasmission. Indeed, recently it was shown that increasing the CSPα dimerization inhibits synaptic transmission [28].

Synaptic dysfunction has been consistently reported in several human and animal models of NCLs [45]. Several NCL-encoded proteins have been found in synaptic compartments [46], [47], [48]. Furthermore, signs of synaptic dysfunction (reduction in synaptic vesicle number) and degeneration have been demonstrated in PPT1 deficient neurons *in vitro* [49], and synaptic pathology (redistribution of SNARE complex and aggregates of Syp/SNAP25) occurs early on in disease progression in the congenital form of NCL [50]. Synaptic involvement in two different mouse models of INCL was also recently demonstrated [45], [51]. The PPT1 null animal model displays alterations in the endocyclic\recycling pathway of synaptic vesicles associated with the impairment of depalmitoylation of the SNARE proteins [45], unlike CSPα null models that do not exhibit any primary defects in the endocytosis process or vesicle recycling [52]. To date, the mechanisms causing synaptic vulnerability in NCLs remains poorly understood.

Our genetic results confirm *DNAJC5* is the disease-causing gene of some ANCLs with autosomal dominant inheritance [13]. The *in silico* analysis suggests reduced the membrane binding and subsequent missorting of CSPα may play a crucial role in the pathogenicity of these mutations. This mislocalization can by itself affect the palmitoylation status and the propensity to aggregate. Thus, a dominant-negative mechanism resulting from CSPα propensity to self aggregate may be involved in the pathogenicity. The mutated CSPα may aggregate with the wild type, induces mislocalization and subsequent reduction of CSPα levels in the synapse

Since CSPα is a synaptic protein and the null animal models show a progressive neurodegenerative phenotype, a better understanding of the cellular and molecular characteristics of synaptic vulnerability will be important for our understanding of NCLs pathogenesis and for the effective development of therapeutic approaches.

## Materials and Methods

### Patients and Study Design

The Institutional Review Board (IRB) at the Washington University School of Medicine in Saint Louis approved the study. Prior to their participation, a written informed consent was reviewed and obtained from family members. The Human Research Protection Office (HRPO) approval number for our ADRC Genetics Core family studies is 93-0006.

Peripheral blood/brain tissue was collected and genomic DNA was isolated from several pathologically confirmed cases (4∶2, 4∶4, 5∶5, 6∶7 and 6∶9) and unaffected samples (5∶2, 5∶11, 6∶1 7∶1, 7∶2, 7∶3, 7∶5 and 7∶6). The mean age at onset for the disease is 36 yr±2.5 (range 32–40). Some of the unaffected samples, such as 5∶2 (70 years old), 5∶11 (65 years old) and 6∶11 (46 years old), are much older than the oldest known AAO (age = 40). In addition, there are skin biopsies showing the absence of the disease. These data indicate that individuals 5.2, 5∶11 and 6∶11 are very unlikely to be a mutation carrier.

To identify the gene underlying disease in this family, the affected individuals 5∶5 and 6∶7 and the unaffected sibling control 5.2 were selected for exome-sequencing. The affected samples are distant enough in the family tree that they share about 1/8 of their genome and since both are affected they should harbor the same single causative variant. The control is an unaffected sibling of one affected sample, which in theory should allow us to reduce the non-pathogenic variants shared by these individuals by ∼50%. By combining the data from these three individuals we were able to narrow down the number of potential pathogenic variants. We also used DNA available from another affected (6∶9) and two unaffected samples (5∶11, 6∶11) to perform an extended segregation analysis.

### Validation Set of patient with ANCLs

There were at least 5 similarly affected individuals in the family over 3 generations with apparent autosomal dominant inheritance. The proband had behavioral difficulties that started in the mid 20 s, probably OCD-like. The first generalized seizure occurred in the early 30 s and was followed by cognitive regression (loss of speech-language and memory). There were also mobility problems by early 30 s.

A patient selected from the second family is 70 years old with a history of tremor, progressive encephalopathy with rectilinear inclusions only (no FP, CL, GRODS) on skin biopsy. NCL gene testing was negative. The daughter has early-adult onset tremors and myoclonic jerks, with rectilinear inclusions in buffy coat. NCL gene testing was negative

Patient has a late-infantile NCL with onset of progressive myoclonic epilepsy at age four, visual loss at age five and curvilinear and fingerprint bodies on nerve/muscle biopsy. This patient is a carrier of a change c.896C>T/P299L in the CLN6 gene [11].

An individual from the third family (two sisters) had childhood absence seizures, followed by the onset in her late 20 s of myoclonus, generalized seizure disorder, and dementia. History of autosomal dominant transmission and electron-microscopical studies revealed an accumulation of dense osmophilic material with a vague internal architecture resembling fingerprint shapes and occasional curvilinear bodies [53].

### Exome sequencing

Enrichment of coding exons and flanking intronic regions was performed using a solution hybrid selection method with the SureSelect® human all exon 50 Mb kit (Agilent Technologies, Santa Clara, California) following the manufacturer's standard protocol. This step was performed by the Genome Technology Access Center at Washington University in St Louis. The captured DNA was sequenced by paired-end reads on the HiSeq 2000 sequencer (Illumina, San Diego, California). Next, raw sequence reads were aligned to the reference genome NCBI 36/hg18 by using Novoalign Version V2.07.00 (Novocraft Technologies, Selangor, Malaysia). Base/SNP calling was perform by SNP Samtools Version 0.1.7. SNP annotation was carried out using version 5.07 of SeattleSeq Annotation server (see URL). [16].

### SNP Genotyping

Two different genotyping technologies were used: MassARRAY SNP (Sequenom, Inc) and KASPar. The principle of the MassARRAY system is PCR-based, where different size products are analyzed by SEQUENOM MALDI-TOF mass spectrometry [17], [18], [19]. The KBioscience Competitive Allele-Specific PCR genotyping system (KASP) is FRET-based endpoint-genotyping technology, v4.0 SNP (KBioscience) [17], [18], [19]. The proportion of SNPs with genotype call rates were >98% and the number of samples that failed to give data for >98% of SNPs was extremely small.

### Sanger Sequencing

Primers were designed using web-based Primer 3 (see URL) (See: Table S2).

Protein-coding exons and 100 base of flanking upstream and downstream intronic sequence of *DNAJC5* (Transcript: ENST00000360864) were amplified on Applied Biosystems (Applied Biosystems, Carlsbad, California, USA) 96-Well GeneAmp® PCR System 9700 Thermal Cyclers using a touchdown protocol. All PCR products to be sequenced were amplified under the same conditions (25-µl volume containing 10× PfuUltra™ HF reaction buffer (Stratagene, La Jolla, California, USA), 5× Betaine (Sigma-aldrich, St Louis, USA), 100 µmol/l each dNTP, 200 nmol/l each primer, 0.4 PfuUltra™ High-Fidelity DNA Polymerase (Promega); PCR profile: 94°C followed by 34 cycles of 45 s at 94°C, 45 s at 62°, and 1 min at 72°C). PCR products purification was completed with Exosap-IT (USB Corporation). Sequencing was performed both in forward and reverse direction using BigDye® Terminator v3.1 Cycle Sequencing Kit (ABI) on an ABI 3130 sequencer. Sequence traces were analyzed using Sequencher (v4.7, Gene Codes Corp, Ann Arbor, Michigan, USA)

### Bioinformatics Analysis

The URLs used for the *in silico* analysis:

Online Mendelian Inheritance in man (OMIM), http://www.omim.org

1000 Genomes, http://www.1000genomes.org/

dbSNP, http://www.ncbi.nlm.nih.gov/projects/SNP/

NCL Mutation Database, http://www.ucl.ac.uk/ncl/

PolyPhen-2, http://genetics.bwh.harvard.edu/pph2/

SIFT, http://sift.jcvi.org/www/SIFT_BLink_submit.html

SeattleSeq Annotation server, http://gvs.gs.washington.edu/SeattleSeqAnnotation

Primer 3, http://frodo.wi.mit.edu/primer3/input.htm

ClustalW2 - Multiple Sequence Alignment,

To analyze the degree of conservation of the predicted amino acid substitutions, multiple sequence alignment was performed using ClustalW [54]. The human (Homo sapiens) CSPα protein sequence (Q9H3Z4) was compared to *Mus musculus, Bos taurus, Rattus norvegicus, Gallus gallus, Xenopus (Silurana) tropicalis and Canis familiaris*.

http://www.ebi.ac.uk/Tools/msa/clustalw2/

### Disease-network analysis of NCLs genes

It has been shown that genes that are associated with phenotypically close disorders are prone to have similar molecular signatures, especially in inherited disease [21]. Here, we have used a disease-network analysis approach as supporting *in silico* evidence of the role of the candidate genes we identified by exome sequencing. We found that the analysis is robust across different algorithms and random subsets of training NCL disease genes.

We have used Endeavour [55], which was trained with all possible features except BLAST. Endeavour has been recently benchmarked using 450 pathway maps and 826 disease marker sets, containing a total of 9911 and 12,432 genes, respectively. It was reported that the area under the receiver operating characteristic curves is 0.97 for pathway and of 0.91 for disease gene sets [56].

Endeavour, http://homes.esat.kuleuven.be/bioiuser/endeavour/tool/endeavourweb.php

ToppGene [57] was used with the default training parameters. Both softwares were trained using the causal genes of other NCLs (NCL Mutation Database, URL) along with genes that are associated with phenotypically close disorders (i. e. differential diagnosis of ANCLs). They were tested against the variants that we have identified by exome sequencing. As a control group we used the entire genome as a training group (See: Table S1).

ToppGene, http://toppgene.cchmc.org/prioritization.jsp

### *In silico* Palmitoylation analysis

We used as positive controls, artificially induced mutations that have experimentally been proven to reduce the palmitoylation status of CSPα such as p.C113-119S, p.C121-124S, p.C121-124A and p.K137A [25], [26].

We used the Clustering and Scoring Strategy for Palmitoylation Sites Prediction (CSS-Palm) system. It has been shown that the program's prediction performance has a highly positive Jack-Knife validation results (sensitivity 82.16% and specificity 83.17% for cut-off score 2.6) [58].

CSS-Palm 3.0 Online Server, http://csspalm.biocuckoo.org/online3.php

### Analysis of the impact of mutations on the hydropathy of CSPα

Protscale, http://web.expasy.org/protscale/

The hydrophobicity profile was calculated using the scale of Kyte and Doolittle [59].

The TMHMM program was used to predict the transmembrane domain in the entire sequence of CSPα (accession number Q9H3Z4). We included the A108-139K residues in the analysis because it has been proven experimentally that this region weakens the membrane affinity of CSPα [25], [26].

TMHMM has shown that it can correctly predict 97–98% of the transmembrane helices. Additionally, it can discriminate between soluble and membrane proteins with both a specificity and sensitivity better than 99% [60].

TMHMM Server v. 2.0, Prediction of transmembrane helices in proteins

http://www.cbs.dtu.dk/services/TMHMM/

Using MPEx, we measured the free energy of transfer from water to a phosphocholine bilayer interface of this domain, ΔG −8.12 for p.KPK137-39del and ΔG −6.97 for p.C121-24L. We also calculated the values of the octanol/interface for these changes; this measure identifies segments that tend to prefer a transbilayer helix conformation relative to an unfolded interfacial location. We found a ΔG of 2.91 kJ/mol for p.C121-124L, ΔG 3.24 kJ/mol for p.KPK137-139del, and ΔG 6.55 kJ/mol for wild type. These two changes in residues increased the membrane affinity of CSPα, in agreement with the experimental findings [25], [26]. Besides the enrichment of hydrophobic residues in the cysteine-string domain, the β-barrel analysis did not find any membrane-spanning strands/regions [61].

The TMX approach uses an experiment-based whole-residue hydropathy scale (WW scale), which includes the backbone constraint, to identify TM helices of membrane proteins with an accuracy greater than 99% [61], [62].

Membrane Protein Explorer (MPEx), MPEx 3.2 server

http://blanco.biomol.uci.edu/mpex/

The SPLIT accuracy is 99% for predicting 178 transmembrane helices in all membrane proteins or subunits of known 3D structure [63].

Split 4.0, Membrane Protein Secondary Structure Prediction Server,

http://split.pmfst.hr/split/4/

### Analysis of conformational changes induced by the mutations

There is only tertiary structural information for the 1–109 residue N-terminal region of mouse homologous CSPα (pdb entry 2CTW; Riken Structural Genomics Initiative), therefore, it was not possible to simulate the effect of the mutations at the structural level.

Prediction of amyloid structure aggregation(PASTA)benchmarked on the dataset of 179 peptides derived from the literature revealed close to an 80% true positive prediction with a ∼20% false positive rate at a PASTA energy threshold of −4.0 [27], [64].

PASTA, http://protein.cribi.unipd.it/pasta/.

Zyggregator method is based on three physico-chemical properties of the polypeptide chain, hydrophobicity, charge, and the propensity to adopt α-helical or β-sheet structures. This method reproduces to a remarkable extent (r = 0.85) the changes in the aggregation rates observed experimentally for single amino acid substitutions [31].

Ztox score uses the same equation as in Zagg but the difference being that the parameters are fitted on a database of polypeptide chains whose aggregation resulted in protofibrillar species, rather than amyloid fibrils [65]. The propensities predicted by Ztox to form protofibrillar aggregates correlate very strongly with their in vivo effects (Stox, r = 0.83) [31].

Zyggregator: Prediction of Protein Aggregation Propensities,

http://www-vendruscolo.ch.cam.ac.uk/zyggregator.php

The folding propensity profile (Z-fold) is defined in terms of the physicochemical properties of the amino acids such as hydrophobicity, secondary-structure propensity, and electrostatic charge [29].

Sequence-Based Prediction of Folding Rates,

http://www-vendruscolo.ch.cam.ac.uk/camfold.php

The CamP method predicts if regions of a structure are protected from hydrogen exchange with an accuracy in the range 80%–100%; The region is protected if lnp>5 for all of its amide groups [32]

Sequence-Based Prediction of Protein Flexibility in Native States

http://www-vendruscolo.ch.cam.ac.uk/camp.new.php

## Supporting Information

Figure S1 **Aggregation profile of CSPα.** A. Aggregation profile of WT CSPα. B. Aggregation profile of p.L115R mutation. C. Aggregation profile of p.L116del mutation D. Aggregation profile of Aβ40 (it is used here as a positive control). This is the output file from PASTA server (see introduction Methods)
(TIF)

Table S1 **Summary of comparison of results from ToppGene and Endeavour gene prioritization analysis.**
(DOC)

Table S2 **Set of primers used for Sanger Sequencing.**
(DOC)
