# Supplementary figures and images for: Exome-Sequencing Confirms DNAJC5 Mutations as Cause of Adult Neuronal Ceroid-Lipofuscinosis
Source: PLoS One. 2011 Nov 4;6(11):e26741. doi: 10.1371/journal.pone.0026741 (PMC3208569; doi:10.1371/journal.pone.0026741)

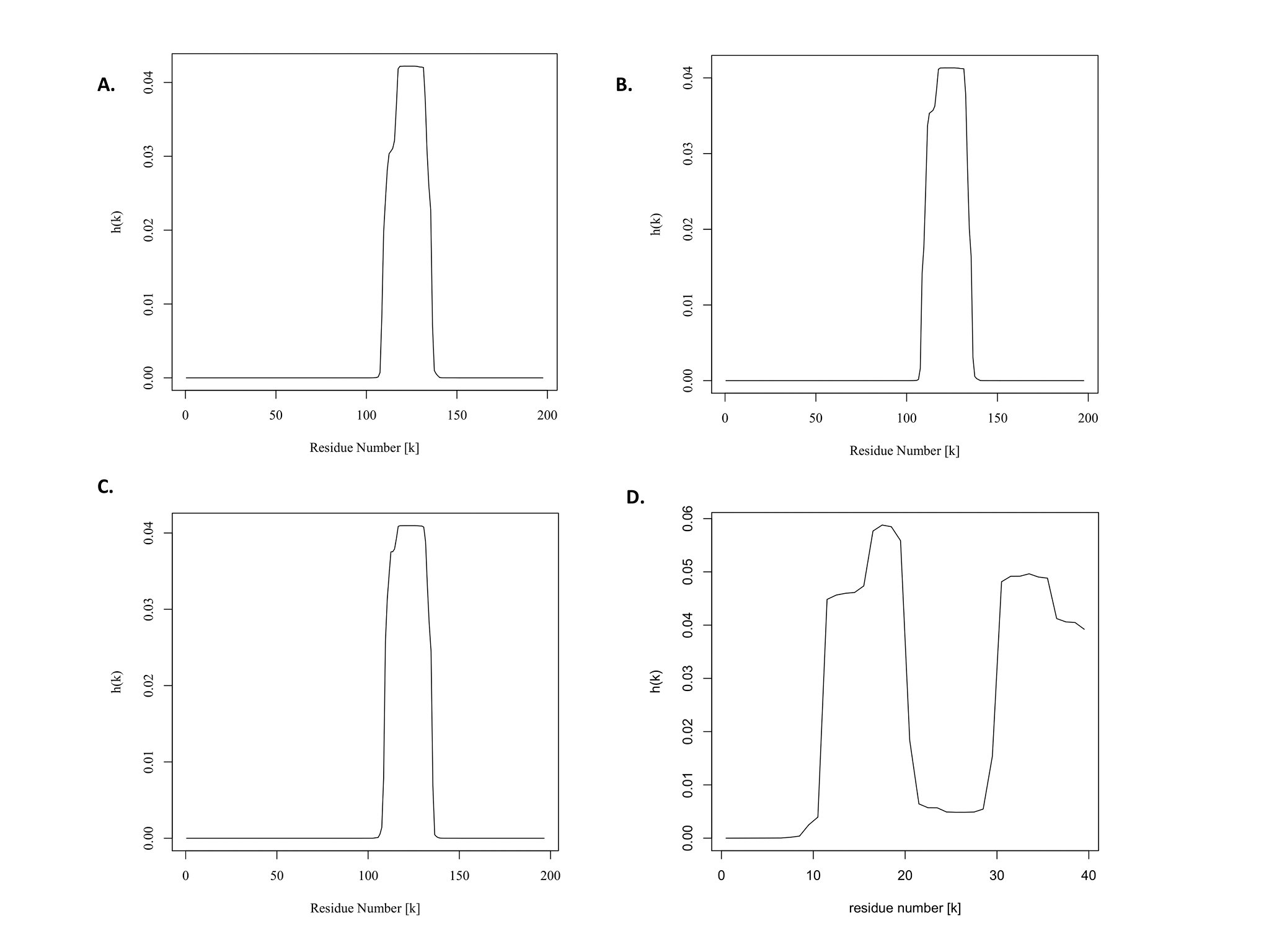

Supplement: Figure S1 — Aggregation profile of CSPα. A. Aggregation profile of WT CSPα. B. Aggregation profile of p.L115R mutation. C. Aggregation profile of p.L116del mutation D. Aggregation profile of Aβ40 (it is used here as a positive control). This is the output file from PASTA server (see introduction Methods) (TIF) [file pone.0026741.s001.tif]
