# Supplementary material for: Exome-Sequencing Confirms DNAJC5 Mutations as Cause of Adult Neuronal Ceroid-Lipofuscinosis
Source: PLoS One. 2011 Nov 4;6(11):e26741. doi: 10.1371/journal.pone.0026741 (PMC3208569; doi:10.1371/journal.pone.0026741)
Supplement: Table S1 — Summary of comparison of results from ToppGene and Endeavour gene prioritization analysis. (DOC) [file pone.0026741.s002.doc]

**Table S**1. Summary of comparison of results from ToppGene and Endeavour gene prioritization analysis

| Gene Symbol | Toppgene Results | | | Endeavour results | | Combined score |
| --- | --- | --- | --- | --- | --- | --- |
|  | Rank Net | Average Score | Overall pValue | Global prioritization score | Rank |  |
| DNAJC5 | 1 | 0.7031 | 7.07E-05 | 2 | 0.117 | 1.5 |
| CDC16 | 3 | 0.4483 | 0.0133049 | 3 | 0.167 | 3 |
| PDCD6IP | 5 | 0.3351 | 0.0246281 | 1 | 0.071 | 3 |
| NPY1R | 2 | 0.6797 | 2.30E-04 | 5 | 0.249 | 3.5 |
| LIPJ | 4 | 0.3063 | 0.015721 | 4 | 0.215 | 4 |
| ZNF717 | 8 | 0.1556 | 0.1493499 | 7 | 0.29 | 7.5 |
| LCMT1 | 10 | 0.1008 | 0.1746657 | 8 | 0.312 | 9 |
| MUC3A | 12 | 0.1445 | 0.2637284 | 6 | 0.256 | 9 |
| ENPP3 | 7 | 0.2334 | 0.1058833 | 13 | 0.531 | 10 |
| ABCC1 | 11 | 0.2423 | 0.2043685 | 9 | 0.337 | 10 |
| ARHGAP10 | 9 | 0.1517 | 0.1615053 | 12 | 0.479 | 10.5 |
| TNRC6A | 6 | 0.3164 | 0.0609277 | 16 | 0.705 | 11 |
| LOC100287879 | 14 | -1 | 0.3513417 | 11 | 0.41 | 12.5 |
| CCDC74B | 15 | -1 | 0.3513417 | 10 | 0.355 | 12.5 |
| TTBK2 | 13 | 0.1311 | 0.2709082 | 15 | 0.689 | 14 |
| LOC389906 | 16 | -1 | 0.3513417 | 14 | 0.69 | 15 |

Both softwares (ToppGene and Endeavour) were trained using the causal genes of other NCL (NCL Mutation Database, URL) plus genes that are associated with differential diagnosis of ANCLs. They were tested against the variants that we have identified by exome sequencing.
