# Supplementary material for: Exome-Sequencing Confirms DNAJC5 Mutations as Cause of Adult Neuronal Ceroid-Lipofuscinosis
Source: PLoS One. 2011 Nov 4;6(11):e26741. doi: 10.1371/journal.pone.0026741 (PMC3208569; doi:10.1371/journal.pone.0026741)
Supplement: Table S2 — Set of primers used for Sanger Sequencing. (DOC) [file pone.0026741.s003.doc]

**Table S**2. Set of primers used for Sanger Sequencing.

| Gene | Primers | Size | Tm |
| --- | --- | --- | --- |
| DNAJC5-Exon2_F | gtggcaaactccacaaggcagtgtt | 365 | 69.43 |
| DNAJC5-Exon2_R | caacgctgagtcggccaggataaag |  | 70.57 |
| DNAJC5-Exon3_F | ctgggtggacctgccttccactg | 594 | 71.27 |
| DNAJC5-Exon3_R | aaccctgcaggcgtggagtgac |  | 70.05 |
| DNAJC5-Exon4_F | ggaaggcagtatccccacctggaac | 400 | 70.46 |
| DNAJC5-Exon4_R | cggcacagtgtcagtgccctcc |  | 71.68 |
| DNAJC5-Exon5_F | tctcccggtggagagtttgtccag | 392 | 69.8 |
| DNAJC5-Exon5_R | CATGCTACGCACTTCGTGGGTCAAG |  | 70.66 |
